# BLV-miR-B1-5p Promotes *Staphylococcus aureus* Adhesion to Mammary Epithelial Cells by Targeting MUC1

**DOI:** 10.3390/ani13243811

**Published:** 2023-12-11

**Authors:** Shuai Lian, Pengfei Liu, Xiao Li, Guanxin Lv, Jiahe Song, Han Zhang, Rui Wu, Di Wang, Jianfa Wang

**Affiliations:** 1 College of Animal Science and Veterinary Medicine, Heilongjiang Bayi Agricultural University, Daqing 163319, China; lianlianshuai@163.com (S.L.); 18311786547@163.com (P.L.); 13359505332@163.com (X.L.); lgxarron@foxmail.com (G.L.); 15764567654@163.com (J.S.); 18746410672@163.com (H.Z.); fuhewu@126.com (R.W.); 2 China Key Laboratory of Bovine Disease Control in Northeast China, Ministry of Agriculture and Rural Affairs, Daqing 163319, China; 3 Heilongjiang Provincial Key Laboratory of Prevention and Control of Bovine Diseases, Daqing 163319, China; 4 College of Biology and Agriculture, Jiamusi University, Jiamusi 154007, China

**Keywords:** dairy cows, BLV, BLV-miR-B1-5p, MUC1, *Staphylococcus aureus*

## Abstract

**Simple Summary:**

Bovine leukemia virus (BLV) is a retrovirus found in cattle, which reduces the lifespan of infected cows and is significantly associated with the occurrence of mastitis in dairy herds. A thorough understanding of the impacts and mechanisms of BLV on the antimicrobial defense function of mammary epithelial cells in dairy cows is essential for preventing and managing bovine mastitis. BLV-encoded microRNAs (BLV-miRNAs) represent functional components that contribute to oncogenesis. This study aimed to examine how BLV-miR-B1-5p promotes *Staphylococcus aureus* (*S. aureus*) adhesion to bovine mammary epithelial (MAC-T) cells via miRNA target gene prediction and validation. The results indicate that BLV-miR-B1-5p promotes *S. aureus* adhesion to bovine mammary epithelial cells by targeting mucin 1 (MUC1).

**Abstract:**

Bovine leukemia virus (BLV) is widely prevalent worldwide and can persistently infect mammary epithelial cells in dairy cows, leading to reduced cellular antimicrobial capacity. BLV-encoded microRNAs (BLV-miRNAs) can modify host genes and promote BLV replication. We previously showed that BLV-miR-B1-5p significantly promoted *Staphylococcus aureus* (*S. aureus*) adhesion to bovine mammary epithelial (MAC-T) cells; however, the pathway responsible for this effect remained unclear. This study aims to examine how BLV-miR-B1-5p promotes *S. aureus* adhesion to MAC-T cells via miRNA target gene prediction and validation. Target site prediction showed that BLV-miR-B1-5p could target the mucin family gene mucin 1 (MUC1). Real-time polymerase chain reaction, immunofluorescence, and dual luciferase reporter assay further confirmed that BLV-miR-B1-5p could target and inhibit the expression of MUC1 in bovine MAC-T cells while interfering with the expression of MUC1 promoted *S. aureus* adhesion to MAC-T cells. These results indicate that BLV-miR-B1-5p promotes *S. aureus* adhesion to mammary epithelial cells by targeting MUC1.

## 1. Introduction

Bovine leukemia virus (BLV) is a retrovirus found in cattle that reduces the lifespan of infected cows [1]. BLV has been largely eradicated from Western European countries [2]; however, infection rates in some Chinese dairy herds are approaching 50% [3]. Notably, many infected cattle show no clinical signs during the long incubation period, which could lead breeders to overlook the infection. However, BLV still affects the function of immune cells [4,5,6], causing reduced immune system function in affected cattle and interfering with the effectiveness of conventional vaccination [7,8]. BLV can continuously infect mammary epithelial cells, thus reducing their antimicrobial capacity, potentially leading to the development of mastitis in dairy cows [9]. BLV loads in the blood of cows with mastitis were shown to be significantly higher than those in healthy cows [10].

BLV-miRNAs as functional elements essential for efficient BLV replication were abundantly expressed in infected cells, accounting for up to 40% of total miRNAs in infected cells, with expression levels of some BLV-miRNAs reaching 80,000 copies/cell [11,12]. It has been shown that BLV failed to induce leukemia after removal of the functional elements of BLV-miRNA transcription [13].

BLV spreads primarily by invading other cells through BLV-loaded lymphocytes, and CAT1/SLC7A1 has been identified as a cellular receptor for BLV infection [14]. After entering the host cells, BLV-miRNAs help BLV to evade the cells’ antiviral immunity by suppressing the expression of key host genes [15]. Thus, BLV-miRNAs may create an environment favorable for lymphocyte migration, persistent BLV replication, and disease development. For example, in bovine B-lymphocytes, the BLV receptor is specifically bound to the surface of the lymphocytes and BLV-miRNAs protect BLV-infected cells from apoptosis by suppressing the expression of host apoptosis-related genes, which facilitates the persistent replication of BLV [13]. In mammary epithelial cell microenvironments, BLV-miRNAs may mediate the synergy between bacterial infection and viral replication to promote the development of mammary inflammation.

MUC1 is a member of the mucin family, expressed by several epithelial tissues, including the mammary gland [16]. Bovine MUC1 protein is synthesized by mammary epithelial cells and has an antimicrobial protective role [17]. MUC1 can attach to bacteria on the cell surface and then sheds from the surface to isolate the bacteria and prevent them from adhering to the cell surface. The preliminary results of this study showed that BLV-miR-B1-5p significantly promoted the adhesion of *S. aureus* to mammary epithelial cells, but whether BLV-miR-B1-5p promotes bacterial adhesion by targeting MUC1 is unclear. A better understanding of the effects and mechanisms of BLV in relation to the antimicrobial defense function of mammary epithelial cells in dairy cows is crucial to prevent and control bovine mastitis. The current study therefore aimed to investigate the mechanism by which BLV-miR-B1-5p promoted *S. aureus* adhesion to mammary epithelial cells, using miRNA target gene prediction and validation.

## 2. Materials and Methods

### 2.1. Cell Culture

Bovine mammary epithelial (MAC-T) cells from Heilongjiang Bayi Agricultural University were cultured in 90% Dulbecco’s Modified Eagle Medium (DMEM)/Nutrient Mixture F-12 with 10% fetal bovine serum (#FSP500, Excell Bio, Guangzhou, China) and 1% penicillin–streptomycin liquid at 37 °C and 5% CO_2_ in a stable environment. After two or three generations of culture when the cells had reached 50–70% confluence, the cells were transferred to well plates for culture. No mycoplasma contamination was detected.

Furthermore, 293T cells were cultured in 90% DMEM with 10% fetal bovine serum, with other conditions consistent with the culture of MAC-T cells.

### 2.2. Cell Transfection (MAC-T)

BLV-miR-B1-5p mimics with the sequence AGGCUGUGGUGGUGGUGCACUGGCU were purchased from Sangon Biotech (Shanghai) Co., Ltd. (Shanghai, China). MAC-T cells were inoculated into six-well culture plates and divided into four groups: 10, 50, and 100 pmol BLV-miR-B1-5p and a negative control (NC, scrambled oligonucleotide) group. When the MAC-T cells reached 60–80% confluence, BLV-miR-B1-5p mimics and NC were transfected with Lipofectamine RNAiMAX Transfection Reagent (#13778150; Invitrogen, Waltham, MA, USA). The medium was changed to a fresh penicillin–streptomycin-free medium from 6 h after transfection until 24 h. We also tested the effect of BLV-miR-B1-5p mimics on MAC-T cells using the CCK8 assay (Figure S1).

### 2.3. Bacterial Adhesion

*S. aureus* was cultured in a prepared Luria–Bertani medium for recovery. Individual colonies were picked for purification for two to three generations, and the cultured *S. aureus* broth was then centrifuged at 5000× *g* for 10 min and the bacteria were collected. DMEM plus 10% fetal bovine serum was added to adjust the bacterial concentration to 10^7^ cfu/mL. After 24 h of transfection with BLV-miR-B1-5p mimics and NC, the cells were washed three times with PBS and 1 mL of diluted bacterial solution was added, followed by incubation for 1 h. The cells were then rinsed thoroughly and fixed with 4% paraformaldehyde for 30 min at 4 °C in the refrigerator. Gram staining was performed and the cells were examined under oil immersion using a light microscope to observe differences in bacterial adhesion. The number of bacteria and number of cells were counted in the same field of view, and the bacterial adhesion rate was calculated as the ratio of adherent bacteria to adherent cells.

### 2.4. Target Gene Binding Site Prediction

The mature sequence AGGCUGUGGUGGUGCACUGGCUU of BLV-miR-B1-5p was obtained by querying miRBase (www.mirbase.org; accessed on 4 August 2022), and the 3′ untranslated region (UTR) sequence of the target gene bovine *MUC1* was searched on the UCSC (genome.ucsc.edu; accessed on 4 August 2022) website. Finally, STarMir was used to pair binding site and target site prediction analyses.

### 2.5. Luciferase Reporter Gene Assay

We carried out a dual luciferase assay to confirm the targeting of BLV-miR-B1-5p to MUC1. BLV-miR-B1-5p binding site wild-type (MUC1-3′UTR-WT) and mutant (MUC1-3′UTR-Mut) plasmids were constructed separately. The purified linearized vector (pSI-Check-2) was ligated with the designed target fragment polymerase chain reaction (PCR) primers using the HB infusion (Hanbio Biotechnology, Shanghai, China) one-step cloning ligation system. Single clones were selected for colony validation and validated clones were selected for sequencing. Samples of correctly sequenced clones were subjected to plasmid extraction. The successfully prepared vector was co-transfected into 293T cells with BLV-miR-B1-5p mimic or NC (three repetitions). According to the Promega Dual-Luciferase system protocol (Madison, WI, USA), transfected 293T cells were lysed using 1× Passive Lysis Buffer and then lysed by centrifugation at 12,000 rpm at 4 °C for 10 min to obtain the supernatant. The supernatant was mixed with Luciferase Assay Reagent II (Promega) working solution in a 96-well plate, and the firefly luciferase value was recorded as the internal reference value. Stop & Glo Reagent (Promega) was then added and mixed well, and the Renilla luciferase value was recorded using a multimode microplate reader as the luminescence value of the reporter gene.

### 2.6. Analysis of Relative Gene Expression

Total RNA was extracted from transfected cells using the TRIzol technique, and cDNA was obtained by employing a PrimeScript RT Reagent Kit with a gDNA Eraser kit (#RR047A; Takara, Kusatsu, Japan). The following primers were designed and synthesized by Sangon Biotech (Shanghai) Co., Ltd. (Shanghai, China) Co. and used for PCR amplification: MUC1 (F: TGCTTACAGTTGCCAATGTCCCTAC and R: GGCTGTGAGTAAGGTGGTAGTTGTG) and ACTB (Internal gene: β-actin gene) (F: GATCTGGCACCACACCTTCTACAAC and R: GATCTGGGTCATCTTCTCACGGTTG). The primers were first pre-deformed for 30 s using TB Green Premix Ex Taq according to the manufacturer’s instructions (RR420A; Takara), followed by 40 PCR cycles of denaturation at 95 °C for 5 s and annealing at 60 °C for 30 s (Bio-Rad CFX Maestro 2.3 system, CFX Connect Thermal Cycler, BioRad, Hercules, CA, USA) (100 ng cDNA was added to the reaction mixture). The relative expression of MUC1 was calculated by the 2^−ΔΔCt^ method.

### 2.7. Immunofluorescence of MAC-T Cells

MAC-T cells from three to four passages of culture were seeded at low density into well plates lined with crawl sheets, and BLV-miR-B1-5p transfection was performed as described above (Section 2.2). After 24 h of transfection, the cells were rinsed thrice with PBS using a shaker for a duration of 5 min each time and then fixed with 4% paraformaldehyde for 30 min at 4 °C. The cells were washed once more and incubated with Triton X-100 at room temperature for 10–15 min to permeabilize the cell membrane. Following rinsing, the cells were blocked with 3% BSA for 30 min, and then incubated with MUC1 antibody (1:250, #4538S; Cell Signaling Technology, Danvers, MA, USA) at 4 °C overnight. After washing the cells every other day, they were incubated with a fluorescent secondary antibody (1:400, #SA00013-1, Proteintech, Rosemont, IL, USA) for 1 h at room temperature in the dark. The cell slides were treated with an anti-fluorescence quenching blocking agent containing DAPI and stored in the dark at 4 °C. The test results were then observed using a laser confocal microscope.

### 2.8. MUC1 Interference

We inhibited MUC1 expression to determine its effect on bacterial adhesion. Three pairs of bovine MUC1 small interfering RNAs (siRNAs) and an NC siRNA were designed by Hanbio Biotechnology (Table 1). The sequences were transfected into MAC-T cells for 48 h at concentrations of 50 and 100 pmol, respectively, as recommended in the product specifications. The optimal interfering sequences and transfection conditions were determined by quantitative reverse transcription PCR. Successful interference with MUC1 expression was verified by Western blot. Bacterial adhesion was then examined in cells transfected with MUC1 siRNA according to the above conditions to determine the effect of MUC1 expression on bacterial adhesion.

### 2.9. Statistical Analysis

The attachment rate was calculated as the number of bacteria divided by the number of cells, ×100%. The results were expressed as the mean ± standard deviation. The data were analyzed using GraphPad Prism 8.0 software, and the statistical significance for measures in Section 3.1, Section 3.2.2, Section 3.3 and Section 3.5 Figure 7A,E, was ascertained using the ANOVA multiple comparison method. The significance for measures in Section 3.5, Figure 7C was analyzed using a Student’s *t*-test. A value of *p* < 0.05 was deemed to indicate statistical significance.

## 3. Results

### 3.1. BLV-miR-B1-5p Promoted S. aureus Adhesion to Mammary Epithelial Cells

**Figure 1:**
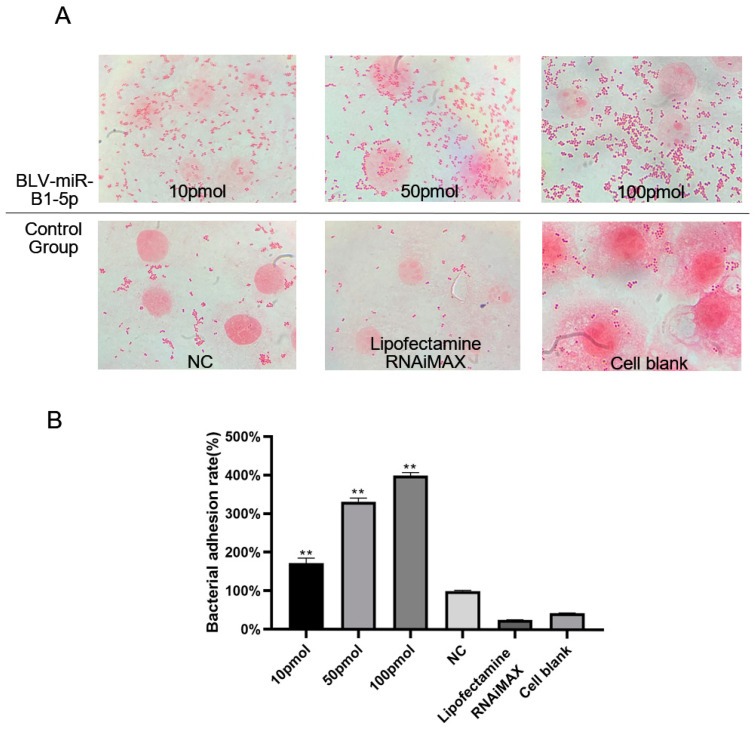
Bacterial adhesion. (**A**) Adhesion of *S. aureus* to MAC-T cells after transfection with different concentrations of BLV-miR-B1-5p. (**B**) Adhesion rates of *S. aureus* to MAC-T cells. Note: Lipofectamine RNAiMAX, transfection reagent control; cell blank, normal cultured cell control; ** *p* < 0.01 compared with NC group.

We determined whether BLV-miR-B1-5p affected the *S. aureus* adhesion to MAC-T cells by transfecting the cells with different concentrations of BLV-miR-B1-5p and NC and co-culturing them with *S. aureus* (Figure 1A). Cells transfected with BLV-miR-B1-5p showed more bacterial adhesion than the NC group, and there were more adhesions in the 100 pmol group compared with the 50 and 10 pmol groups (171.75% (±13.46%), 331.25% (±10.38%), and 399.22% (±8.17%), respectively). The transfection of BLV-miR-B1-5p significantly promoted *S. aureus* adhesion to MAC-T cells (*p* < 0.05, Figure 1B).

### 3.2. MUC1 as a Target of BLV-miR-B1-5p

#### 3.2.1. Target Site Prediction

**Figure 2:**
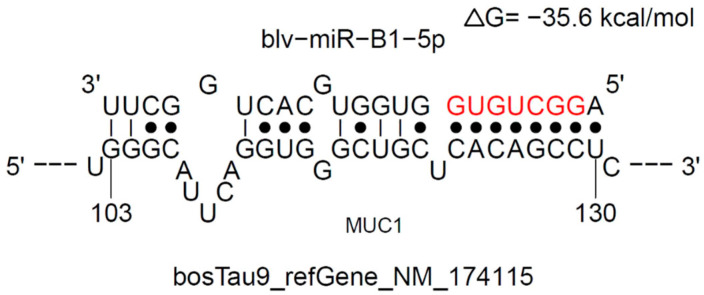
Prediction of BLV-miR-B1-5p duplexes with MUC1 transcripts using the STarMir program. BLV-miR-B1-5p seed region in red.

The BLV-miR-B1-5p binding site on MUC1 was identified by STarMir (https://sfold.wadsworth.org/cgi-bin/starmir.pl (accessed on 7 May 2022) (Figure 2). The free degree of binding was −35.6 kcal/mol, which was less than the theoretical binding value of −15 kcal/mol [18], suggesting that BLV-miR-B1-5p may target MUC1 at this site.

#### 3.2.2. Dual Luciferase Assay

**Figure 3:**
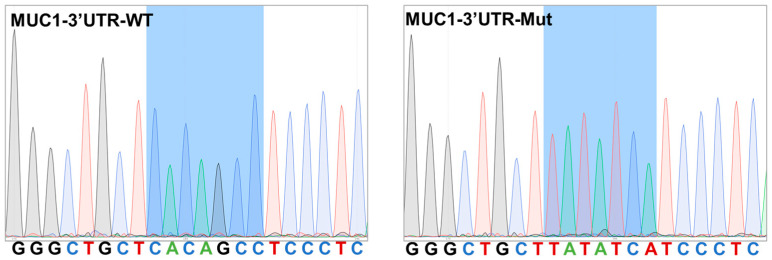
Verification of the mutants by Sanger sequencing.

The sequencing results of both MUC1-3′UTR-WT and MUC1-3′UTR-Mut plasmids indicated successful construction of the mutant plasmids (Figure 3). The manufacturer’s instructions were followed during amplification of the bacteria and purification of the plasmid.

**Figure 4:**
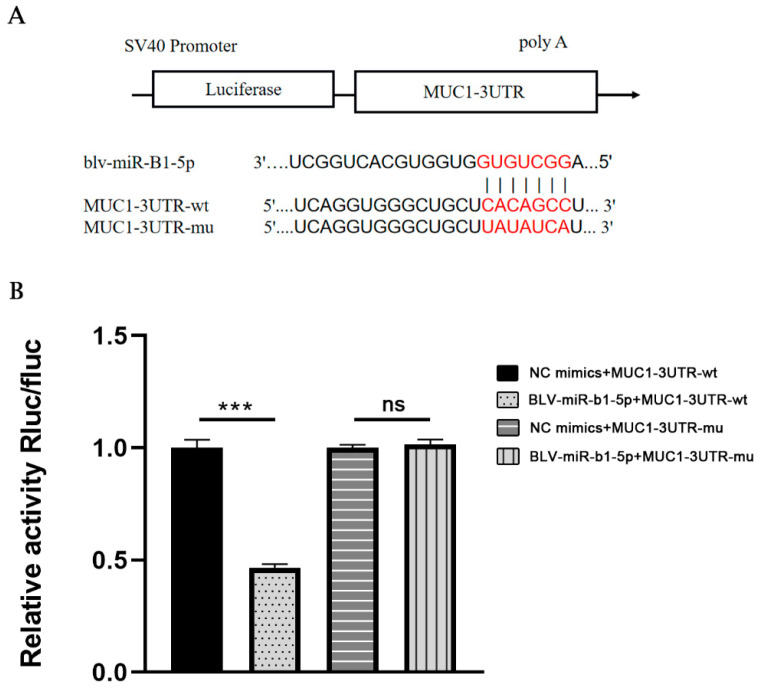
Dual luciferase reporter target gene assay. (**A**) Schematic diagram of BLV-miR-B1-5p binding to MUC1-3′UTR target site (BLV-miR-B1-5p seed region in red). (**B**) Dual luciferase reporter gene assay for BLV-miR-B1-5p interaction with MUC1-3′UTR. Note: *** *p* < 0.001; ns, no significant difference.

The constructed vector was co-transfected into 293T cells with BLV-miR-B1-5p or NC, and the resulting luciferase activity was measured. A schematic diagram of BLV-miR-B1-5p binding to the MUC1-3′UTR target site is shown in Figure 4. BLV-miR-B1-5p significantly downregulated the expression of luciferase in MUC1-3′UTR-WT cells compared with the NC group (*p* < 0.001, Figure 4B), while BLV-miR-B1-5p failed to downregulate the expression of luciferase in the MUC1-3′UTR-MUT compared with the NC group (*p* > 0.05), indicating that BLV-miR-B1-5p targeted MUC1.

### 3.3. BLV-miR-B1-5p Repressed MUC1 Gene Expression

**Figure 5:**
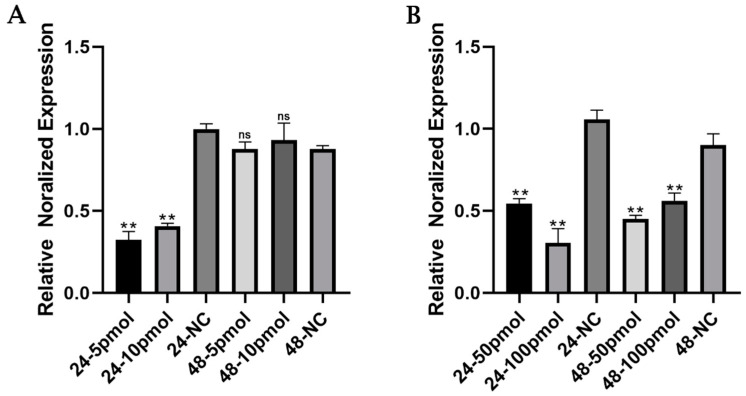
Effects of different concentrations of BLV-miR-B1-5p on relative expression levels of MUC1 mRNA after treatment of MAC-T cells for 24 and 48 h, respectively. (**A**) Low-concentration treatment group, (**B**) high-concentration treatment group. Note: ** *p* < 0.01 compared with NC group; ns, no significant difference compared with NC group.

MUC1 mRNA expression was highly significantly downregulated in bovine mammary epithelial cells transfected with different concentrations of BLV-miR-B1-5p for 24 h (*p* < 0.001) in the low concentration group (Figure 5A). MUC1 mRNA expression was also highly significantly downregulated (*p* < 0.01) in both the 50 and 100 pmol groups at 24 and 48 h, with the most significant downregulation (*p* < 0.001) in the 100 pmol group at 24 h (Figure 5B). These results indicated that BLV-miR-B1-5p inhibited the expression of MUC1 in MAC-T cells. Subsequent experiments were performed in cells transfected for 24 h with 10, 50, and 100 pmol BLV-miR-B1-5p, respectively.

### 3.4. BLV-miR-B1-5p Repressed MUC1 Protein Expression

**Figure 6:**
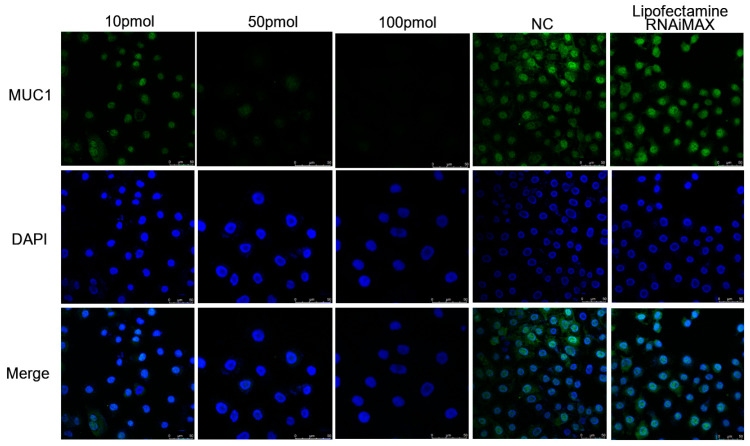
Effect of transfection of MAC-T cells with different concentrations of BLV-miR-B1-5p on MUC1 protein expression. (The nucleus was labelled in blue, the MUC1 protein was labelled in green).

After the transfection of MAC-T cells with different concentrations of BLV-miR-B1-5p for 24 h according to the above conditions, the immunofluorescence results showed that BLV-miR-B1-5p has the potential to decrease the fluorescence intensity of the MUC1 protein (Figure 6), indicating that BLV-miR-b1-5p inhibited the expression of the MUC1 protein in MAC-T cells.

### 3.5. Silencing of MUC1 Promoted S. aureus Adhesion to Mammary Epithelial Cells

**Figure 7:**
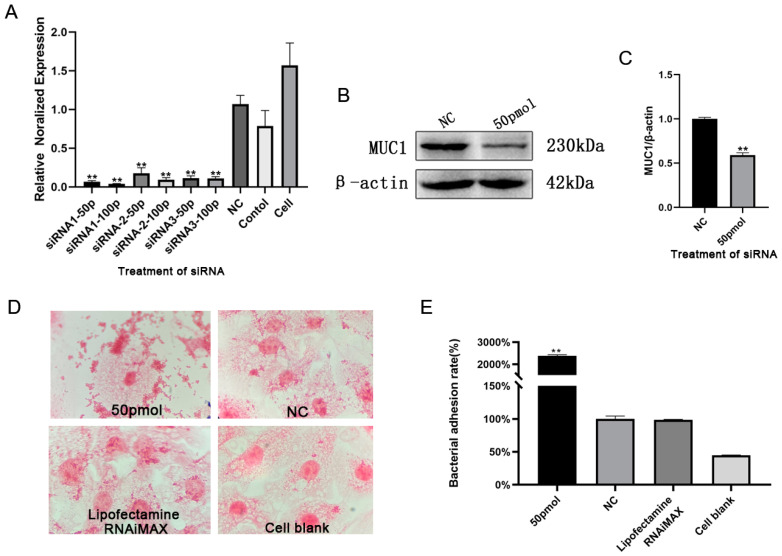
Effects of MUC1 RNA interference in bovine mammary epithelial cells. Effects of interference on (**A**) MUC1 fluorescence quantification in mammary epithelial cells, (**B**) MUC1 protein expression in mammary epithelial cells, (**C**) MUC1/β-actin grayscale analysis, (**D**) bacterial adhesion, and (**E**) *S. aureus* adherence rate. Note: ** *p* < 0.01 compared with NC group.

To confirm if the differential bacterial adhesion was related to MUC1, three pairs of MUC1-interfering sequences were synthesized and transfected at concentrations of 50 and 100 pmol for 48 h. All three pairs of interfering sequences significantly reduced MUC1 mRNA expression levels (Figure 7A). Transfection with siRNA1 50 pmol for 48 h was selected for subsequent experiments. MUC1 protein expression was significantly reduced after transfection with 50 pmol MUC1 siRNA (Figure 7B,C). The Western blot results for transfection with other concentrations of siRNA1 are shown in the supplementary file (Figure S2). Furthermore, interfering with MUC1 expression significantly promoted the adhesion of *S. aureus* to bovine mammary epithelial cells (Figure 7D,E).

## 4. Discussion

miRNAs are small noncoding RNAs that target and regulate endogenous genes and act as major post-transcriptional regulators of gene expression [19]. A significant number of miRNAs have been identified in both the mammary gland and milk of dairy cows, with their expression patterns being greatly influenced by the physiology of the cow and the type of pathogen present [20]. BLV has been reported to alter the mRNA and miRNA profile of small extracellular vesicles in milk [21,22,23,24]. BLV can also encode BLV-miRNAs (BLV-miR-B1, B2, B3, B4, B5) [11,12], which are functional elements that contribute to oncogenesis in vivo [13], and the ablation of BLV-miRNAs affects BLV replication and suppresses leukemia development [25].

Virus-encoded miRNAs that target host genes within host cells can affect various intracellular functions, including immunity, autophagy, and apoptotic signaling pathway transduction, thereby acting on the viral pathogenic process. For example, miR-US25-1-5p encoded by human cytomegalovirus (HCMV) targets CD147 mRNA, thereby inhibiting the body’s natural antiviral immune function [26]. Virus-encoded miRNAs have been shown to suppress the host’s antiviral immune function by binding to host genes. Virus-encoded miRNAs also protect cells from DNA damage and apoptosis caused by viral infection, e.g., human herpesvirus-8 miR-K9 reduced the expression of cleaved caspase-3 and caspase-7 [27] and HCMV-encoded miR-UL70-3p inhibited apoptosis by suppressing expression of the pro-apoptotic gene *MOAP1* [28]. BLV-miRNAs also alter host genes and promote viral replication and pathogenesis [29]. In addition, BLV-miRNAs have been suggested to be involved not only in tumorigenesis, but also in cell signaling, apoptosis, and immunity [30]. Casas et al. suggested that BLV-miR-B1-5p targeted genes associated with stress response and immune response processes and was also involved in the development of human leukemia [31]. The finding of the current study suggested that BLV-miR-B1-5p could promote *S. aureus* adhesion to MAC-T cells; however, its role in the promotion of bacterial internalization requires further exploration.

A study by Sando et al. confirmed that bovine MUC1 may be an inducible innate immune effector that acts as an important first line of defense against bacterial invasion of the epithelial cell surface, especially in mammary epithelial cells and neonatal intestine components [17]. MUC1 in mammals also protects the epithelial cell surface from invasive pathogenic microorganisms [32,33,34]. The gastrointestinal tract of MUC1-deficient mice was shown to be more susceptible to colonization by Helicobacter pylori, thus supporting the role of MUC1 in protecting epithelial tissues from bacterial infection [35]. Prior to validating the target genes, it was postulated that BLV-miR-B1-5p would interact with the *MUC1* gene, as confirmed by dual luciferase experiments. Moreover, transfection with different concentrations of BLV-miR-B1-5p significantly downregulated MUC1 gene and protein expression levels, indicating that BLV-miR-B1-5p could target and inhibit the expression of MUC1. Meanwhile, transfection with BLV-miR-B1-5p also significantly increased the adhesion between *S. aureus* and MAC-T cells. However, it was not clear if this phenomenon was related to BLV-miR-B1-5p targeting of MUC1. We therefore transfected cells with MUC1 siRNAs to inhibit MUC1 expression and repeated the bacterial adhesion experiments. Notably, bacterial adhesion was significantly increased following MUC1 interference compared with the control group, indicating that BLV-miR-B1-5p promoted the adhesion of *S. aureus* to MAC-T cells by targeting MUC1.

**Figure A1:**
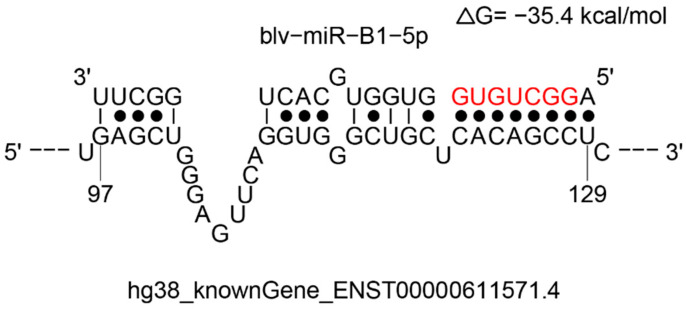
Prediction of BLV-miR-B1-5p duplexes with human MUC1 transcripts using the STarMir program.

BLV-miR-B1-5p may also act on intestinal epithelial cells, thus increasing the potential for intestinal invasion by pathogenic microorganisms. In addition to targeting bovine MUC1, we also considered that BLV-miR-B1-5p might target human MUC1, and the prediction results accordingly showed that the binding site of BLV-miR-B1-5p to human MUC1 was highly consistent with that for bovine MUC1 (Appendix A: Figure A1). Further studies are needed to assess the safety risks of BLV-miRNA regulation of human genes.

## 5. Conclusions

In summary, BLV-miR-B1-5p promotes the adhesion of *S. aureus* to mammary epithelial cells by targeting MUC1. This may be one of the pathways by which BLV promotes the development of mastitis in dairy cows.

## Figures and Tables

**Table 1:** siRNA sequence information.

Name	Sequence
siRNA1	F: GCUGUUCCCAGUGCUUACA TT
	R: UGUAAGCACUGGGAACAGC TT
siRNA2	F: GAAGCACACGCAGCCAGUUAU
	R: AUAACUGGCUGCGUGUGCUUC TT
siRNA3	F: GCCACUUCCGCCAACUUGUAA TT
	R: UUACAAGUUGGCGGAAGUGC TT
siRNA NC	F: UUCUCCGAACGUGUCACGU TT
	R: ACGUGACACGUUCGGAGAA TT

## Data Availability

The data presented in this study are available on request from the corresponding author.

## Supplementary Materials

The following supporting information can be downloaded at: https://www.mdpi.com/article/10.3390/ani13243811/s1, Figure S1: The CCK-8 results; Figure S2: Effect of different concentrations of siRNA1 on MUC1 protein expression.
