# Supplementary material for: BLV-miR-B1-5p Promotes Staphylococcus aureus Adhesion to Mammary Epithelial Cells by Targeting MUC1
Source: Animals (Basel). 2023 Dec 11;13(24):3811. doi: 10.3390/ani13243811 (PMC10741194; doi:10.3390/ani13243811)
Supplement: Supplementary file 1 [file animals-13-03811-s001.zip › animals-2686785-supplementary.pdf]

Supplementary Results

(1) CCK-8 assay was applied to detect the viability of MAC-T cells with different concentrations of BLV-miR-B1-5p mimics. As shown in Figure S1, there were no significant differences between the groups (*P*>0.05).

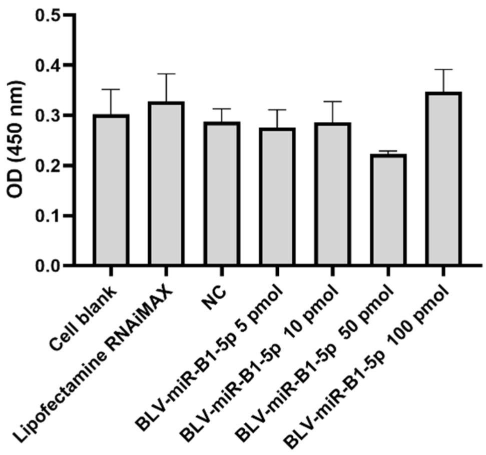

Figure S1. The CCK-8 results. OD, optical density.

(2) As shown in Figure S2, siRNA1 at 10 pmol, 50 pmol and 100 pmol could all inhibit the expression of MUC1 protein.

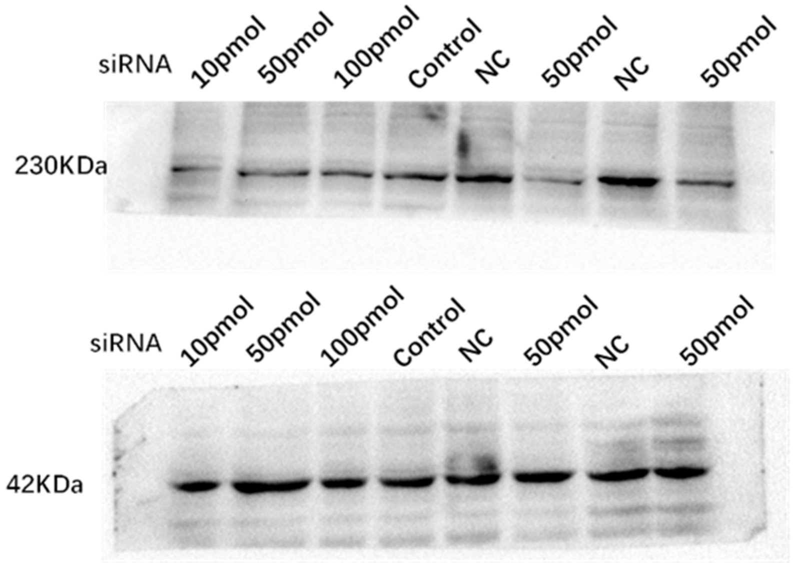

Figure S2. Effect of different concentrations of siRNA1 on MUC1 protein expression.
